# Temperature Increase Limitation During Automatic Bone Drilling in Orthopedic Surgery

**DOI:** 10.7150/ijms.136882

**Published:** 2026-09-20

**Authors:** Tony Boiadjiev, George Boiadjiev, Ivan Chavdarov, Kamen Delchev, Rumen Kastelov

**Affiliations:** 1 Department of Distributed Information and Control Systems, Institute of Information and Communication Technologies, Bulgarian Academy of Sciences, 1113 Sofia, Bulgaria.; 2 Department of Mechatronics, Robotics and Mechanics, Faculty of Mathematics and Informatics, University of Sofia “St. Kliment Ohridski”, 1504 Sofia, Bulgariа.; 3 Institute of Robotics, Bulgarian Academy of Sciences, Acad. G. Bonchev St, Bl. 1, 1113 Sofia, Bulgaria.; 4 Center of competence “Smart Mechatronic, Eco- and Energy Saving Systems and Technologies”, Acad. G. Bonchev Str, Bl. 2, 1113 Sofia, Bulgaria.; 5 Orthopedic and Trauma Clinical Centre of Ministry of Domestic Affairs, 1000 Sofia, Bulgaria.

**Keywords:** automatic bone drilling, temperature increase control, drill speed control

## Abstract

In orthopedic surgery, the most performed manipulation is bone drilling. One significant problem related to this manipulation is the overheating which causes thermal osteonecrosis. By reports the drill speed is the main factor responsible for heat generation and contributes up to 78% to temperature rise during bone drilling. Therefore, the aim of this work is to develop and implement drilling speed control approach to control the temperature rise in drilling area and prevent overheating of bone tissue. During drilling, a real-time drill bit tip temperature estimation is calculated based on data from a non-contact temperature sensor and information from the robot control system. This way, temperature feedback during drilling process is realized. Further, on the base of modified PI controller the target speed depending on the increase in the drill bit tip temperature is calculated. Applying the drill speed control approach, the experimental results showed a decrease in the maximum temperature on the drill bit tip during drilling by up to 13 °C was observed, and the temperature rise was limited to 65-70 °C, with values above 65 °C lasting less than 2 s. The novelty of this work is the realization of temperature feedback and drill speed control approach during drilling process. Applying them in a robotic bone drilling process, the temperature increase in the instrument-bone area was significantly limited, the critical temperature values at which thermal osteonecrosis occurs were not reached, and that substantially reduces the likelihood of thermal osteonecrosis during drilling.

## Introduction

Thermal osteonecrosis of bone tissue is one of the most serious problems that can occur during bone drilling manipulation. Thermal osteonecrosis refers to the death of bone tissue due to extremely high temperatures, hinders bone regeneration and can have a negative outcome regarding to fracture fixation by implants in bones 1 and can even lead to loosening or breakage of bone screws 2.

Thermal osteonecrosis depends on the maximal temperature of the bone tissue during drilling, as well as duration of heating, i.e., on drilling time interval. Bone cell death with respect to heat occurs immediately at 70 °C 1, as well as necrosis of cells occurs at heat expose to 50 °C for 5 s. Drilling cortical bone with a thickness of about 5 mm, depending on the feed rate, may require a time of 10 s at a feed rate of 0.5 mm/s, or 5 s at a feed rate of 1 mm/s. Increasing the feed rate reduces the drilling time interval, so the risk of thermal osteonecrosis becomes less. However, increasing in feed rate causes an increase in the thrust force and the risk of traumatic osteonecrosis becomes higher. The drilling process is characterized by a set of input and output parameters, respectively 31 and 8 in number according to 3. The input parameters define the conditions, under which the manipulation is performed, and the output parameters determine its output responses in bone drilling. For each specific manipulation, the input parameters, except for feed rate and drill speed, are known in advance. The feed rate and the drill speed have the greatest influence on the output parameters. According to statistics, the most critical output response parameters are temperature and applied forces in the drilling process 3, since they are decisive in terms of the probability of occurrence of traumatic and thermal osteonecrosis in the “bone - drill bit” area. Thermal and mechanical damage to the bone affects the contact area and cell growth between the bone and the screw, which is the main cause of pullout strength implant reduction and failed fixation 2. This circumstance also explains the numerous scientific studies and developments related to the influence of these parameters on the quality of the drilling process. When it comes to thermal osteonecrosis of bone tissue drill speed is the main factor, compared to other input parameters, responsible for heat generation. Drill speed affects proportionally the maximal temperature of bone drilling 4, as drill speed increases, the generated heat increases 5,6 and contributes to 78.87% 7, 51.8 % 8, and 61% 9 to temperature rise during drilling.

When conducting experimental research *in vitro*, the temperature during drilling is measured in two ways - using thermocouple and using thermal infrared (IR) camera. The thermal camera only measures the temperature on the surface of the object (bone) - there is no information about the temperature inside the bone in the drilling area. The thermocouples are pre-implanted in the bone at a certain distance and at a certain depth from the point where the drilling should actually execute. Placing thermocouples within the bone, adjacent to the drilling surface, as a means of measuring temperature is not a satisfactory solution to the problem of temperature measurement due to the insulating effect of the bone itself and that if temperatures greater than 45 ºC are reached by this method, then the temperature at the drill-bit cutting edge must be considerably higher with consequent severe damage to the bone surface of the hole 10. It can be concluded that both methods of temperature measurement are practically inapplicable during real bone drilling performance in surgical room (*in vivo*). Due to the problems associated with real time temperature feedback realization in the conditions of bone drilling manipulation in surgical room there are no scientific developments related to controlling the drilling process based on drilling area temperature information.

Numerous scientific studies are known related to the experimental evaluation of the influence of the separate input parameters of the drilling process on the temperature increase in the drilling zone. Several thermo-mechanical models have been published 11-14 to predict heat generation; the temperature rise and thermal injury in bone during a drilling process. The presented thermal models are used for sensitivity analysis in order to reveal the effect of input parameters on output parameters and for parametric study to determine optimal cutting conditions and drill bit geometries, which reduce thermal damage in the bone drilling process simulation. The complex relationships between the drilling conditions, drill-bit geometry, and bone characteristics present a major challenge in determining the optimal set of bone drilling parameters to simultaneously minimize thermal and traumatic bone damage.

The control of the cutting conditions (drilling speed and feed rate) that determine the thrust force and temperature in the drilling area during drilling is the main factor to obtain an optimal outcome. This can be achieved by implementing force and temperature feedback during drilling in the conditions of the robotic bone drilling process. Force feedback is realized through a force sensor integrated into the mechanical system of the robot. The temperature feedback during bone drilling process can be realized by using a temperature sensor positioned in a suitable place so that it does not interfere the manipulation in real conditions.

Throughout the drilling process, the feed rate and drill speed values can change. In manual drilling, the surgeon controls the feed rate (respectively applied or thrust forces) based on his individual skills. However, simultaneous control of the feed rate and drill speed in manual execution is practically impossible to achieve. This can be realized in robotic execution by the robot control system depending on its capabilities. Due to the ability to control feed rate and drill speed during drilling the opinion is confirmed that the quality of manipulations increases with the participation of assisting robots.

The application of traditional robots in surgical practice is hindered by their high cost, the need for trained personnel, large footprint in the operating room, and workflow complexity. Their functioning is also associated with procedures such as preoperative planning and registration, which further increase the time required to perform the operation.

As a suitable alternative to traditional robots are the recently appeared handheld robotic systems which are relatively cheap, compact, lightweight and easy to operate and have almost all the capabilities of large multifunctional robots 15-19. A handheld robot is a manipulator, either in parallel, serial or hybrid structure, where the base is held by the human operator 18.

The research presented in this work was carried out using such a robot 20,21. Drill speed has the most significant influence on the value of the maximal temperature during drilling, so the aim of the present study is to develop and implement a drill speed control during drilling that would limit the temperature rise at the tool-bone interface and, accordingly, the risk of overheating and thermal osteonecrosis.

## Materials and Methods

### A. Basis of the Mathematical Model

The temperature estimation and temperature feedback methodology are based on the energy conversion in the bone-robot system, preserving the overall energy balance according to the universal energy conservation law.

The method serves to model the dynamics of hybrid systems, i.e., such systems that receive, transform and release energy of different types - for example, electrical, mechanical, and thermal, etc. The energy interactions taking place within a type of energy are described with terms appropriate for this type - electrical phenomena are described with the terms voltage and current, which correspond to certain mathematical symbols. These symbols are considered the “base variables” of a given energy space, namely what they describe and where this type of energy is considered. This way, different energy spaces related to the type of energy under consideration can be defined. In Table 1, for each “base variables” pair, its physical dimension is given together with the corresponding dimension in terms of the basic units in the SI system - mass M, length L, time T and Ampere I. Historically, each pair of basis variables has been defined by the common terms “Across” and “Through” 22. Common to the bases of all spaces is the property that the product of their basis variables has power dimension [L2M/T3], i.e., the change in the energy per unit of time (for vector variables their scalar product must be understood). This way, an arbitrary system that exchanges energy with its surroundings during its functioning can be considered together with its accompanying energy space, which in turn is composed of as many energy subspaces as there are types of energy that it transforms - both internally between its subsystems, possibly having a different physical nature, and with the external environment. In the further considerations, we will limit ourselves to the energy interaction between mechanical and thermal phenomena, and the energy converted in this process can be considered as external - for example, brought in by the robot's engines.

The table is not limited and can be prolonged for another physical phenomena where similar pair of variables for them can be specified but always their product will have the dimension of power and after integration by the time - the energy. In the next for our purpose, we will concentrate our considerations only on the third, fourth and sixth row of the table where the interaction of mechanical and the thermal energy is described.

In bone drilling process, a relatively much amount of work, done by mechanical subsystem of the robot, is converted directly into heat in the “bone - drill bit” area 1. As a result, the temperature of the drill bit and the bone rise simultaneously. During drilling, the drill heats up much faster than the bone - due to its smaller (from 2.5 to 3 times) specific heat capacity, and the maximal temperature is reached at the upper cutting edge of the drill.

Before starting the drilling, the orthopedic drill is in a state of thermal equilibrium, i.e., the temperature in the separate areas of the drill bit is the same. After starting the drilling, a temperature difference occurs between the tip of the bit (which is heated) and the rest of the bit, and the drill bit goes into a new state. In a body that is not in temperature equilibrium (there is a different temperature in different areas of the body), heat energy is transferring. Such a process is called heat conduction. A quantitative characteristic of thermal conductivity is the heat flow - a vector with a direction of heat transfer and a magnitude determining the intensity of heat transfer.

The present work is related to the publication of the authors 23 as the results here not only build on the previous ones, but are completely new in a qualitative and quantitative sense. In 23 the following estimation was derived for the temperature at the drill bit tip and its relationship to the measured temperature on the drill at a given distance from the contact point with the bone


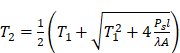
 (1)

where:


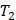
- drill bit tip temperature


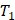
- temperature measured on the drill at a given distance from the point of contact with the bone


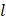
- length between the tip of the drill bit and the measuring point [m]


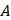
- cross-sectional area of the drill bit [m^2^]


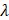
- coefficient of thermal conductivity of the drill bit [W.m^-1^. K^-1^];


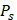
- mechanical power (which enters from both motors) in the bone drilling process

In equation (1), the values of 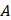
and 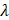
are known in advance, 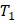
is temperature measured in real time by the temperature sensor in area of the drill outside the drilled bone, 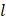
and 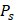
are calculated values based on the information available in the ODRO control system. This makes it possible to calculate the temperature value at the tip of the bit 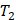, which allows realizing temperature feedback during the bone drilling process. The control of drill speed in the drilling process is based on temperature feedback in accordance with expression (1).

An algorithm had been implemented to calculate the target speed depending on the increase in the temperature of the drill bit tip. The goal of the algorithm was to limit the increase in the temperature of the drill bit tip, i.e., to limit the difference between the current temperature value 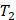, calculated based on expression (1), and a set reference temperature value 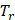. Limiting the increase in the temperature 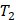
means also limiting the heating of the bone substance, i.e., minimizing the risk of thermal osteonecrosis. The control algorithm is a modified PI controller (2), which is used to calculate the value of the correction 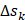
by which the drill speed should be changed in the next sampling cycle:


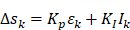
 (2)

where:


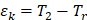
,


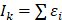
, the summation index varies in the range 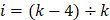,


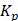
и 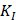
- feedback coefficients of the proportional and integral components of the control, respectively, 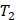
- the current temperature value, 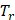
- reference temperature value, 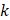
- the current sampling rate. The adjustment of the values of the feedback coefficients was performed experimentally.

The integral component of the control 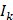
is formed within a sliding window having dimension 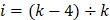, which provides information about the temperature change of the drill tip in the drilling area, where 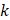
is the current sampling rate. The sliding window components contain the measured and recorded values of the four sampling cycles before the last sliding window component, which contains the value from the current sampling cycle.

The new speed value for the next sampling cycle 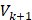
is:


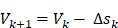
 (3)

where if 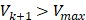
then 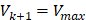
and if 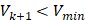
then 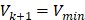. Here 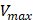
and 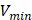
are the maximum and minimum speed values set during drilling respectively, and 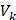
is the speed in the current sampling cycle.

Thus, presented drill speed control algorithm for automatic bone drilling consists of three steps described by (1), (2) and (3).

When setting the reference temperature value 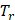, the following is taken into account:

When the temperature of the drill tip reaches 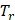, the drilling process continues, i.e., heat generation continues in the drilling zone, and the temperature 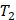
continues to increase.

During the drilling, the temperature of the drill tip 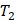
is higher than the temperature to which the bone substance is heated, with the difference between the two temperatures reaching 20 °C 23,24.

The conditions under which thermal osteonecrosis of bone tissue occurs are the temperature exceeding 70 °C of the bone, or the temperature exceeding 50° - 55 °C for more than 5 s 25.

The increase or decrease of 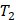
is a relatively inert process, i.e., changes in the values of the parameters under which the drilling process occurs do not lead to immediate changes in the temperature values. Therefore, it is advisable to set the reference temperature value 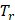, within 45° - 50 °C. The goal is to limit the maximal temperature of the drill tip 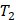
during the drilling process to the range of 65° - 70 °C, which guarantees limiting the bone temperature below 50 °C, which eliminates the possibility of thermal osteonecrosis. At the same time, the following should be taken into account: drilling with a lower drill speed value increases the thrust force 6 and the risk of traumatic osteonecrosis, i.e., the use of low drill speed values should be avoided and applied only when necessary. It should also be noted that the temperature 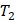
calculated based on expression (1) represents the drill-bit tip upper limit temperature 23. This means the temperature that the drill-bit tip actually reaches during the drilling process is lower than the calculated one, and the bone is actually heated to lower temperatures.

The temperature 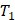
was measured on the drill at a given distance from the point of contact with the bone to minimize the influence of blood flow and bone debris on the measured values.

The presented experimental results were obtained *in vitro*. When performing the manipulation *in vivo*, the blood supply to the bone will undoubtedly have a cooling effect, i.e., the maximal drill bit tip temperature would have a smaller value (compared to drilling *in vitro*). This would lead to a decrease in the value of the heat flow through the drill and, accordingly, to the registration of smaller values of the measured temperature 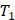
- temperature measured on the drill. This, in turn, would lead to smaller values of the temperature 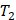, calculated based on expression (1). However, this will not change the logic and execution of the drilling speed control algorithm.

### B. Specimens

The experimental samples were fresh porcine femur bones obtained from licensed meat shops which are state-approved and freely available in markets. From the cross-section in the middle of the diaphysis (Fig. 1), it is seen that the thickness of the bone cortex varies within the range of 2 - 5 mm. All experimental drilling was carried out in this area.

The pig bones are considered as a model for human bones and are similar concerning thermal and mechanical properties, for example: human bone density 2100 [Kg/m3]; pig bone density 2010 [Kg/m3]; human bone specific heat 1260 [J/Kg K]; pig bone specific heat 1330 [J/kg K]; human bone thermal conductivity 0.38 [W/m K]; pig bone thermal conductivity 0.31 [W/m K] 6, 24, 26. Therefore, when conducting *in vitro* experiments, the use of pig bones is perceived as an adequate substitute for human bones.

The control groups represent porcine femur bone samples drilled in the area in the middle of the diaphysis, where the cortical thickness is in the range of 2-5 mm, without cooling system, under condition of robotized bone drilling, using 3- and 4-mm drills with varying degrees of wear. The number of repeated experiments for any condition is 3. The purpose of the executed experiments was to compare the obtained results concerning the rise temperature limitation in the drilling area with and without drill speed control under the equal values of the process input parameters.

### C. Experimental setup

The experiments were performed by the handheld robot ODRO 20, 21 - Fig. 2(A). Actual temperature data during the bone drilling process were obtained from an Impac Pirometer IN 510 temperature sensors 23 with temperature range -40…700 ºC, resolution 1/10ºC, sampling rate 20 ms, repeatability 0,5 ºC. Drill bit material - stainless steel, emissivity 60%. The sensor is equipped with a pyrometer optical head type with additional close focus lens. A schematic illustration of the experimental setup is presented in Fig. 3. The sensor head was positioned to measure the temperature in the area of the drill bit 10 mm from the point of contact with the bone - Fig. 2(B), Fig. 3. The purpose of this positioning was to avoid the influence of bone debris during drilling on the data for the measured temperature 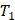. Based on the sensor data, the estimated temperature value of the tip of the drill bit 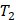
was calculated from the expression (1).

### D. Conditions of the experiment

When executing drilling in “Cortex I” or “Cortex II” mode 20, the drill bit translational movement automatically stops after drilling the first or the second cortex. Maximal drill bit penetration into the soft tissue is within 1 mm. When drilling automatically stops, the display shows the size of the bone cortex walls and marrow. The experiments were performed under the following conditions.

Cutting condition:

Maximal Drill speed - 500 RPM;

Minimal Drill speed - 350 RPM


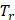
- reference temperature value - 45 ºC

Feed rate: max drilling feed rate - 2mm/s or 4 mm/s 21

Cortical thickness of porcine femur: 2-5 mm.

Drill bit: 3 or 4 mm new or used standard surgical stainless two flute drill bit.

Initial temperature: 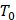
is between 22 - 25 ºC.

The minimal drill speed is the one to which the maximal speed can be reduced as a result of the described speed control algorithm. The measurements were executed in a room where the bone temperature was in average 23 ºC. The initial temperature of drill bit and a bone is not important factor and during the drilling process it increases both in bone and in drill bit from 22 ºC (in room) to temperature of body (37 ºC). That doesn't change the bone properties and doesn't influence the maximal temperature elevation 27.

The experiments are executed without cooling systems. The usage of the cooling systems during the process will doubtless limit the temperature arise. The experiments' purpose is to see and verify the proposed approach ability for the temperature rise limitation in the drilling area without dependence of existing or not of such systems.

## Results

To calculate the drill bit tip temperature value 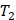, it is necessary to measure the temperature 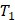
in real time from the additional temperature sensor in a certain area of the orthopedic drill outside the drilled bone, as well as to calculate
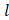
and 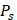
based on the information available in the ODRO control system. The feed rate controller, based on the Trinamic Motion Control Module TMCM 1110 21, as well as a force sensor inside the ODRO, captures required sets of data (force sensor data, position value, feed rate value). The drill speed control was realized on the base of TMCM 1630. The current through the motor during drilling is about 3000 mA, which, considering the torque constant and the gearhead, means a torque of about 1.16 Nm. In the figures, the maximal motor speed was 7000 RPM, which, after a 14:1 gearhead, means a drill speed of 500 RPM. The minimal motor speed was 5000 RPM, which, after a 14:1 gearhead, means a drill speed of 350 RPM.

The experiments were performed in automatic mode Cortex I (Near cortex) for unicortical drilling. After completing the drilling of the corresponding cortex, the motors automatically stop and the drill bit is withdrawn to the starting position. After drilling the display presents the result of operation - the thickness of the near cortex (cortex I) 20.

The results presented in the figures were obtained in real time using TMCL-IDE (Trinamic Motion Control Language - Integrated Development Environment) version 3.5.0.0. - specialized software for programming and working with TRINAMIC motion controllers. In this environment, the results for the selected parameters of the monitored process are displayed in real time in graphical form.

The following notations are valid for all figures presented:

Red line - temperature 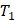
in ºC, measured in real time by the temperature sensor.

Blue line - temperature 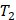
in ºC, calculated on the base of expression (1).

Green line - BLDC motor speed in RPM, scaled by a factor of 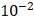.

Brown line - the difference 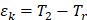
in ºC.

In the figures, the speed of the BLDC motor is scaled by a factor of 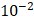, the goal being that the values are comparable and can be represented on a single graph.

We introduce the following designations (specification) for the orthopedic drill bits used in the experiments:

- New orthopedic drill bits (NDB) - used 1 to 5 times

- Slightly used (SUDB) - 6 to 15 times

- Used (UDB) - 16 to 25 times

- Very used (VUDB) - 26 to 50 times

- Worn (WDB) - over 51 times.

The graphs present results obtained at unicortical bone drilling. For all experimental results presented below, the motor starts when contact of the drill bit with the bone was made. Then, within 2 s, a center with a depth of 1 mm was formed at feed rate 0.5 mm/s to avoid possible slipping of the drill bit on the bone. Further, drilling continues at selected feed rate (2 mm/s or 4 mm/s) until the cortex was completely drilled, after which the motors automatically stop and drill speed becomes 0.

On the graphs in the next figures the coordinate axes are the time and the temperature correspondingly.

The results presented in Fig. 4 and Fig. 5 aim to track the maximal temperature when drilling without and with drill speed control and when using NDB 4mm and feed rate of 2mm/s. The drilling process duration was 4 s. The cortex thickness was 4.8 mm.

In both graphs, the difference 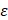
becomes greater than zero approximately 500 ms after the center is formed. Then, in Fig. 5, a reduction in speed is observed as a result of calculating the corrections according to the algorithm already described. The value of the calculated corrections depends on the magnitude of difference 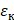. Larger values of difference ε lead to larger values of corrections to the speed. The speed adjustment from Maximal Drill speed to Minimal Drill speed is performed for 1000 ms at a feed rate of 2 mm/s for 2 mm drilling depth. As a result, for Fig. 5 at the end of the drilling process the difference 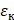
was equal to 11ºC and 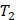
reached a maximum value of 56 ºC.

For Fig. 4 without drill speed control these values are 23ºC and 68ºC respectively. Or there is a limitation on the increase of 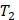
by 12 ºC. At these temperature values, there is no risk of overheating of the bone tissue.

The results presented in Fig. 6 and Fig. 7 aim to track the maximal temperature when drilling without and with drill speed control and when using NDB 3mm and feed rate of 2mm/s. The drilling process duration was 3.4 s. The cortex thickness is 3.6 mm.

In both graphs, the difference 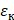
becomes greater than zero approximately 600 ms after the center is formed. Then, in Fig. 7, a reduction in speed is observed. At the end of the drilling process, the drill speed does not reach the Minimum Drill speed and is equal to 6134 RPM. The value of the calculated corrections depends on the size of difference 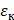. The speed correction is performed within 700 ms. At a feed rate of 2 mm/s - for a drilling depth of 1.4 mm. As a result, for Fig. 7 at the end of the drilling process the difference 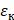
was equal to 6 ºC and 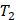
reached a value of 51 ºC. For Fig. 6 without drill speed control these values are 10 ºC and 55 ºC, respectively. Or there is a limitation on the increase of 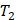
by 4 ºC. At these temperature values, there is no risk of overheating of the bone tissue. Compared to the results in Fig. 4 and Fig. 5, these values are significantly lower, which is due to two factors - the smaller diameter of the NDB used, and the smaller value of cortex thickness, respectively, the smaller drilling time interval, or heat time duration.

The results presented in Fig. 8 and Fig. 9 aim to track the maximum temperature when drilling without and with drill speed control and when using UDB 3mm and feed rate of 2mm/s. The drilling process duration was 5.1 s. The cortex thickness is 5.0 mm for Fig. 8 and 5.1 mm for Fig. 9.

In both graphs, the difference 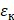
becomes greater than zero approximately 600 ms after the center is formed. Then in Fig. 9 a reduction of the speed is observed. The speed correction from Maximal Drill speed to Minimum Drill speed is carried out for 800 ms. At a feed rate of 2 mm/s - for 1.6 mm drilling depth. As a result of this in Fig. 9 at the end of the drilling process the difference 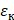
was equal to 27 ºC and 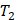
reached a maximum value of 72 ºC. For Fig. 8 without drill speed control these values are 40 ºC and 85 ºC respectively. Or there is a limitation on the increase of 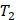
by 13 ºC.

From Fig. 8 it can be seen that values of 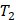
above 65 °C have a duration of about 2 seconds, while values above 70 °C have a duration of 1.4 seconds, therefore there is serious risk of overheating of the bone tissue. From Fig. 9 it can be seen that values of
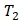
above 70 °C have a duration of 0.3 seconds, while those above 65 °C have a duration of less than 2 seconds - about 1.4 seconds, therefore there is no risk of overheating of the bone tissue.

The results presented in Fig. 10 and Fig. 11 aim to track the maximal temperature when drilling without and with drill speed control and when using VUDB 3 mm and feed rate of 4mm/s.

For Fig. 10 the drilling process duration was 3.1 s. The cortex thickness is 3.8 mm. The temperature 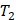
reached 70 ºC with a trend for subsequent increase when drilling a cortex with a greater thickness, which leads to a risk of overheating of bone tissue.

For Fig. 11 the drilling process duration was 3.4 s. The cortex thickness is 4.4 mm. The speed correction from Maximal Drill speed to Minimum Drill speed on the Fig. 11 is carried out for 900 ms. As a result of this for Fig. 11 at the end of the drilling process the difference ε was equal to 18 ºC and 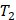
reached a maximum value of 63 ºC. For Fig.10 without drill speed control these values are 25 ºC and 70 ºC respectively. Or there is a limitation of the increase of 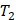
by 7 ºC. At these temperature values for the Fig. 11, there is no risk of overheating of the bone tissue.

The results presented in Fig. 12 aim to track the maximum temperature when drilling with drill speed control and when using worn 3mm drill bit. The reduction of motor speed to 5000 RPM did not lead to effective limitation of the growth of 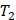, as at the end of the process 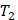
reached 73 ºC. The thickness of the cortex in this case is 3.5 mm, and with a greater thickness the increase in 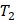, would be even more significant, which would lead to a real risk of thermal osteonecrosis. Under these conditions, the drill speed control algorithm is unable to limit the temperature increase. Therefore, the use of such drills in the operating room is highly undesirable.

A number of experiments were performed at different values of the input parameters feed rate, drill bit diameter, drill bit degree of wear, drilling depth. The obtained results are presented in Table 2. The risk of thermal osteonecrosis is shown in the last column. Data from rows 1 - 9 in the table correspond to the results of graphs 4 - 12. The pairs of rows 1-2, 3-4, 5-6 and 7-8 present the results without and with drill speed control under the same remaining conditions.

The following findings were established:

- for all combinations of input parameters, a limitation of the increase in drill bit tip temperature value 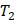
during drilling is observed under drill speed control conditions compared to drilling without drill speed control.

- the difference between the maximal drill bit tip temperature value 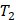
under conditions of drilling speed control and without drilling speed control varies between 4 and 13 °C, the temperature rise 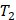
was limited to 65-70 °C, with values above 65 °C last less than 2 s.

- the smallest difference of 4 °C is recorded when drilling with NDB with a smaller diameter and a smaller drilling depth - Fig. 6 and Fig. 7, and this difference increases when using SUDB and UDB and reaches 13 °C in Fig. 8, Fig. 9.

- at a lower feed rate, the drilling time increases, and when using UDB, the risk of overheating the bone tissue increases.

- when using WDB the drill speed control is unable to limit the temperature rise to safe limits, therefore the use of such drills in the operating room is highly undesirable - Fig. 12.

The drill speed control generates speed corrections only when the difference 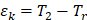
becomes greater than 0. The greater this difference, the greater the speed corrections and the faster the maximal drill speed decreases to the minimum drill speed - Fig. 12. Accordingly, the smaller this difference, the smaller the speed corrections and the slower the maximal drill speed decreases to the minimal drill speed - Fig. 5. At small values of this difference, the corrected drill speed values may not even reach the minimal drill speed - Fig. 7. Or the quick response of the algorithm depends on the specific drilling conditions, respectively on the increase in drill bit tip temperature value 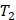
during drilling, with the reduction of the speed from maximal drill speed to minimal drill speed being realized only when necessary. Drilling at a lower drilling speed increases the thrust force and therefore the risk of traumatic osteonecrosis. Therefore, the use of low drilling speeds in the drilling process should be applied only when necessary.

Finally, it can be concluded that by using the drilling speed control approach, the temperature rise in the drilling area is significantly limited. This is clearly seen from the presented experimental results, concerning both the calculated temperature 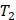
and the really measured temperature 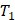.

## Discussion

This study presents development and implementation of drill speed control during automated bone drilling. The experimental results showed the performance of the bone drilling process with and without drill speed control. The results also showed that the applied control algorithm influences and limits the temperature increase in the bone-tool interface area. All that reduces the likelihood of thermal osteonecrosis during drilling.

When commenting, interpreting and analyzing these results, the following should be kept in mind:

In expression (1) 23, the assumption is made by which all mechanical work is converted into heat. It must be noted the energy balance of the process is simplified. For instance, the mechanical energy for overcoming the friction of drill bit in the drilled hole is neglected. It is the same for the heat flow which goes from the drill surface via the drilled hole walls to the bone, and for the heat flow from the drill surface outside the bone to the atmosphere. Using a simplified energy model reduces the number of real-time calculations. But the value of calculated 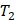
which is obtained using this simplified model, is always greater or equal than the real temperature of the drill bit tip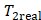, i.e., 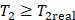. The accordance between the calculated
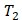
and the real temperature of the drill bit tip 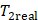
was experimentally verified in 23 using an additional temperature sensor positioned to measure the temperature of the drill bit tip immediately after the breakthrough of the first or second cortex.

In bone drilling a significant amount which is done by mechanical work is converted directly into heat in the bone-drill bit area 28. The temperature of a material which can be reached is related to its coefficients of heat capacity and thermal conductivity 29. The specific heat capacity of the drill bit is up to 3 times smaller than the bone 30. And the drill bit temperature increases much faster than the bone, with the temperature of the drill bit tip being significantly higher than that of the bone. The maximal temperature during bone drilling process appears at the cutting edge of the drill bit 24. The difference of the bone temperature and the drill bit during drilling may reach up to 20 ºC 24.

By the authors knowledge none of studies up to now related to temperature during drilling are developed *in vivo*, therefore the exact temperature for death of the human bone tissue is unknown 29-32. As the level which points the start of thermal osteonecrosis is still not clear and it is under discussion in the scientific publications 1, 25, 33-35, this should be taken into account when determining the safe temperature threshold of drill tip heating (for example 45 °C), at which the control of drilling conditions of the drilling process to avoid thermal osteonecrosis of bone tissue should start. There is agreement on the thesis that the death of bone cells regarding the heat occurs at 70 °C 1,21,34. Results are reported that: a lower temperature than 55 °C for 30 s may cause irreversible bone cell death 35; necrosis of cells occurs at heat expose to 50 °C for 5 s, but this is not true for cells exposed to 50 °C for 2 s 24; recovery is reported in the cells exposed to 50 °C and 60 °C for 2s 25; irreversible damage to bone cells appears when they are exposed to temperatures over 53 °C or 62 °C for 10 s, regardless of the used drilling method 36.

Considering the above, it can be concluded that if the increase in temperature of the drill bit tip 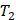
is limited to 65-70 °C, with values above 65 °C lasting less than 2 s, then it can be argued that the bone temperature in the drilling zone will be limited to values that exclude the possibility of thermal osteonecrosis. The experimental results show that this is successfully achieved by the applied drill speed control approach.

The temperature rise also depends on feed rate. The feed rate contributes up to 26% to temperature rise 9 during drilling. The experiments were performed at a feed rate of 2 and 4mm/s. The feed rate of 4mm/s reduces the duration of heating, but increases in the thrust force. In manual drilling in the orthopedic practice mostly the speeds in the range of 350 - 800 RPM are used. The temperature rise is proportional of the value of the drill speed. Because of that the drilling process starts with the speed of 500 rpm. Also, the motor speed reduction was limited to 5000 RPM (350 RPM - ODRO). Drill speed affects proportionally the maximal temperature of bone drilling and from the viewpoint of limiting the temperature rise, the drill speed should be reduced as much as possible 5. It is recommended to keep the drill speed at low values between 50 and 370 rpm 36. However, drilling with a lower drilling speed value increases the thrust force 6 and, therefore, the risk of traumatic osteonecrosis, i.e., the use of excessively low drilling speed values in the drilling process should be avoided and applied only when necessary.

It should also be noted that when new drills are used, and when the cortex thickness is within 2-3 mm, the temperature 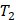
in some cases does not exceed the set temperature 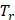
- reference value, and accordingly no correction is observed for drill speed. When the cortex thickness is 3-5 mm, the algorithm makes appropriate adjustments to the drill speed, and the temperature
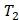
is maintained within safe limits. When worn drill bits are used the temperature 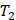
rises above the critical 70 °C, regardless of drill speed adjustments. This is especially true in areas where the cortex thickness is within 4-5 mm. Therefore, the use of such drills in the operating room is highly undesirable. Findings suggest that drills should be replaced after 15 cycles (use and sterilization) to ensure efficacy and patient safety 37.

The experiments presented were performed without the use of coolant. The traditional cooling supply methods (external irrigation) in bone drilling continuously apply coolant, which can obstruct the surgeon's vision. In a real *in vivo* environment, saline, blood, and bone debris can affect the emissivity of the drill surface and the optical path of the sensor, which can make non-contact measurement difficult. However, the use of a suction system removes the debris and coolant, providing a better view of the surgical site for the surgeon and a clean surgical field.

The external irrigation cools only the part of the drill outside the bone, and a surface of the bone, not the deeper layers. The part of the drill in the bone could be cooled only indirectly with lower decrease of bone temperature at the cutting tip 38. This is especially important for the outer cortex that cannot be reached by external irrigation 39, and the heat is accumulated together with the hole depth prolongation and obtains its maximal value when the drill bit exits the hole 25. In internal irrigation, the cooling agent is delivered directly to the point of contact of the cutting edge of the drill and the bone, where a temperature increase occurs due to friction 39.

Although modern orthopedics and bone and joint surgery regularly use external irrigation for cooling of the bone and drill, for orthopedic and traumatology use the open type of internal irrigation is the most efficient parameter for lowering the increase in bone temperature. With its use any other combination of other drill and drilling parameters is of no importance and any of these could be used 38. However, internal irrigation uses specially constructed, open type internally cooled medical steel drills. Drilling is performed with the specially designed hand-held drill with a reception system for open type internally cooled drill 39.

External irrigation is essential for reducing temperature rise, but cannot guarantee that the temperature will be maintained within safe limits 40. The application of the approach presented in this study allows significant limitation of temperature rise in the drilling area. Using this approach in combination with cooling systems will lead to even better results and represents an additional opportunity for preventing the occurrence of thermal osteonecrosis.

The presented experimental results refer to cortical bone drilling. During the osteosynthesis of hip fractures, it is necessary to drill the cancellous bone. Bone density of the cortical bone (1830 Kg/m^3^) is an order of magnitude greater than cancellous bone density (170 Kg/m^3^) 41. Cancellous bone thermal conductivity 0.087 [W/m K] is much smaller than cortical bone thermal conductivity 0.452 [W/m K] 41. This difference in thermal and mechanical properties of the cortical bone and cancellous bone determines the different reaction of the two types of bone regarding the increase in temperature during drilling. Cortical bone, which is the most compact and dense part, generates the highest frictional heat during drilling 27. The increase in temperature when drilling cancellous bone is significantly smaller and does not pose a real risk of thermal damage to the bone tissue.

The limitations of the presented study are related to several aspects: drilling objects - using pig bones instead of human bones; the experiments were performed *in vitro*; experiments were not conducted with all possible variations of the drilling process input parameters such as feed rate, drill speed, and drill bit design.

The pig bones are considered as a model for human bones and are similar concerning thermal and mechanical properties 6,24,26.

Performing experiments *in vivo* requires the availability of appropriate clinical conditions, which the authors do not have. *In vitro* conditions are perceived as a sufficiently good alternative when performing experimental activities in the practical verification of achieved theoretical results. The lack of blood perfusion in the *in vitro* experiments contributes to an additional increase in temperature during drilling. The use of cooling systems during the drilling process, as well as blood perfusion in *in vivo* conditions, will undoubtedly limit the temperature increase but cannot guarantee that the temperature will be maintained within safe limits.

The experiments used 3mm and 4mm drill bits, new and used, 2 mm/s and 4 mm/s feed rate, 3-5mm cortical thickness. This does not cover the possible variations of the input parameters at all. The influence of variations in input parameters on process output parameters, including temperature rise in the drilling zone, has been the subject of numerous studies and is well known and extensively documented. Regardless of the specific values of the input parameters and their possible variations, any bone drilling process is characterized by heat generation, which leads to an increase in the temperature of both the drill bit, as well as bone. Different input parameters contribute to different degrees to the amount of this heat generated, for example: drill diameter 13%; feed rate 26%; drill speed 61% 9. The increase in temperature is proportional to the drill speed value. For this reason, in our work we have focused on drill speed control as the factor that most strongly affects the increase in bone temperature in the drilling zone. The present study presents temperature rise limitation at drilling area by drilling speed control during automated bone drilling.

It should be noted that the density of the drilled bones also has an effect on the temperature. It is also in connection of realizing a predetermined depth of the hole or “smooth” breakthrough from the bone. Information about the density of the drilled bone is obtained in the ODRO control system by an identification procedure 21. Thus, not only the age, gender and health status of the object is known according the information of the bone density, but also of the specific bone being drilled. An additional advantage of this identification is preventing the drill bit from slipping on the bone and distorting the direction of the desired hole, especially when drilling the second cortex. So, the drilling mode automatically takes into account the influence of the density of the drilled bone, in particular on the temperature.

The presented approach is based on the drill bit tip real time temperature estimation and its functioning does not depend on possible variations in the input parameters. It allows additional prevention of the probability of thermal osteonecrosis.

The presented drill speed control approach effectively limits the temperature rise at the tool-bone interface when using drill bits with up to 20 cycles of use and sterilization. However, the same cannot be said when using worn drill bits. In addition, presented drill speed control can be applied in practice only in the conditions of robotic bone drilling.

In the authors' previous work, a computational approach was developed to estimate the upper limit of the drill bit tip temperature in the drilling area. This allows realizing temperature feedback during real time control of bone drilling process using a standard temperature sensor. The accordance between the calculated and the real temperature of the drill bit tip was experimentally validated using an additional temperature sensor positioned to measure the temperature of the drill bit tip immediately after the breakthrough of the first or second cortex.

Current study presents the temperature rise limitation during automated bone drilling in orthopedic surgery via drill speed control. The main contribution of this work is the realization of temperature feedback and drill speed control during bone drilling process. Drill speed control approach was developed to calculate the target speed during drilling depending on the increase in the temperature of the drill bit tip. As a result, the temperature rise in the drilling area is significantly limited.

## Conclusion

This study presents the temperature rise limitation via drill speed control on the base of temperature feedback during automated bone drilling in orthopedic surgery. The temperature feedback was realized using a standard non-contact temperature sensor.

Drill speed control approach was developed to calculate the target speed during drilling depending on the increase in the temperature of the drill bit tip. The drill tip temperature estimation was calculated based on the data obtained from the sensor as well as the information available in the robot control system.

From the presented experimental results, it can be concluded that in automatic bone drilling via the implementation of the drill speed control approach the temperature rise at the tool-bone area during drilling was significantly limited, the critical temperature quantities for thermal osteonecrosis are not reached and that substantially reduces the likelihood of thermal osteonecrosis during drilling.

## Figures and Tables

**Figure 1:**
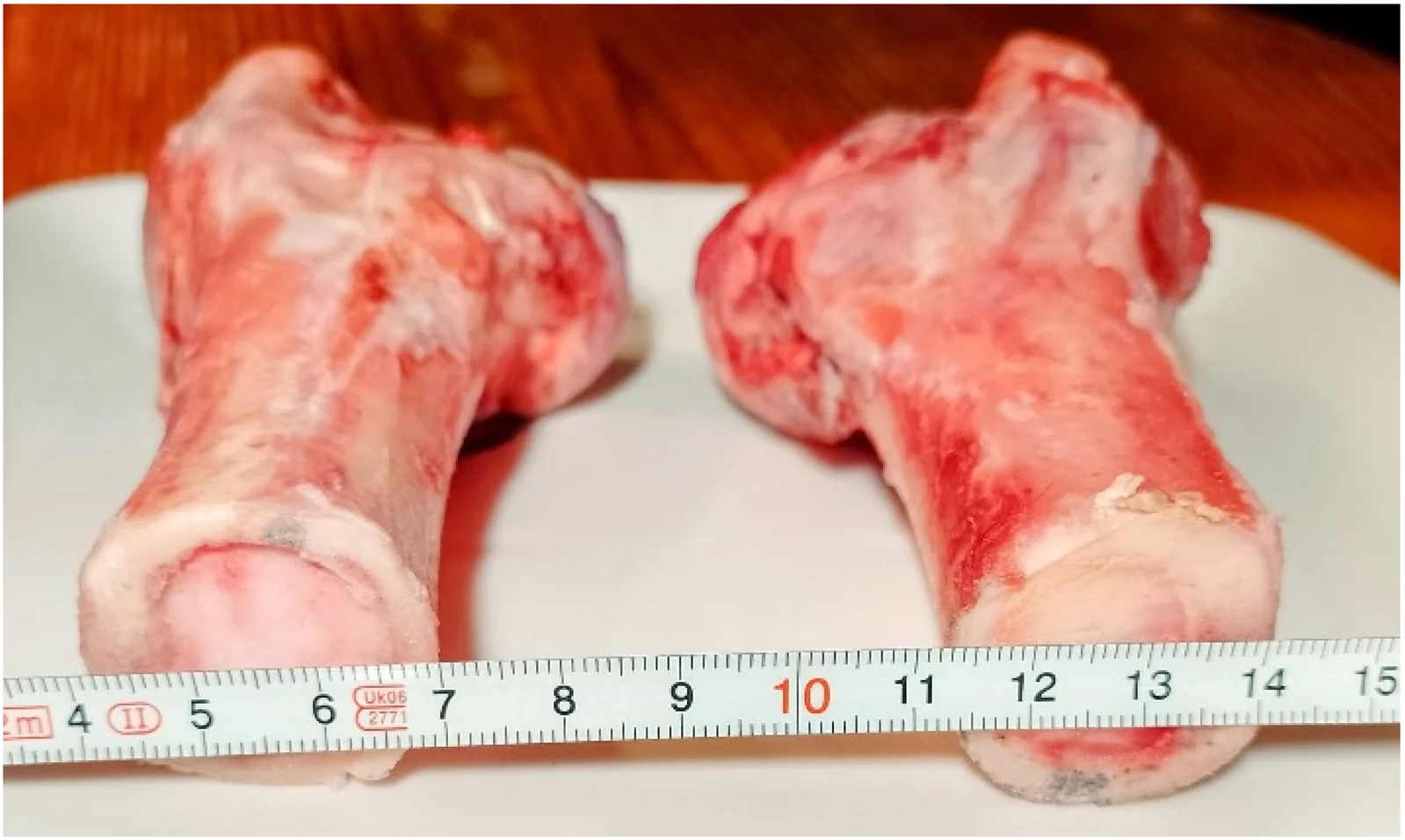
Cross-section in the middle of the diaphysis of the porcine bone samples.

**Figure 2:**
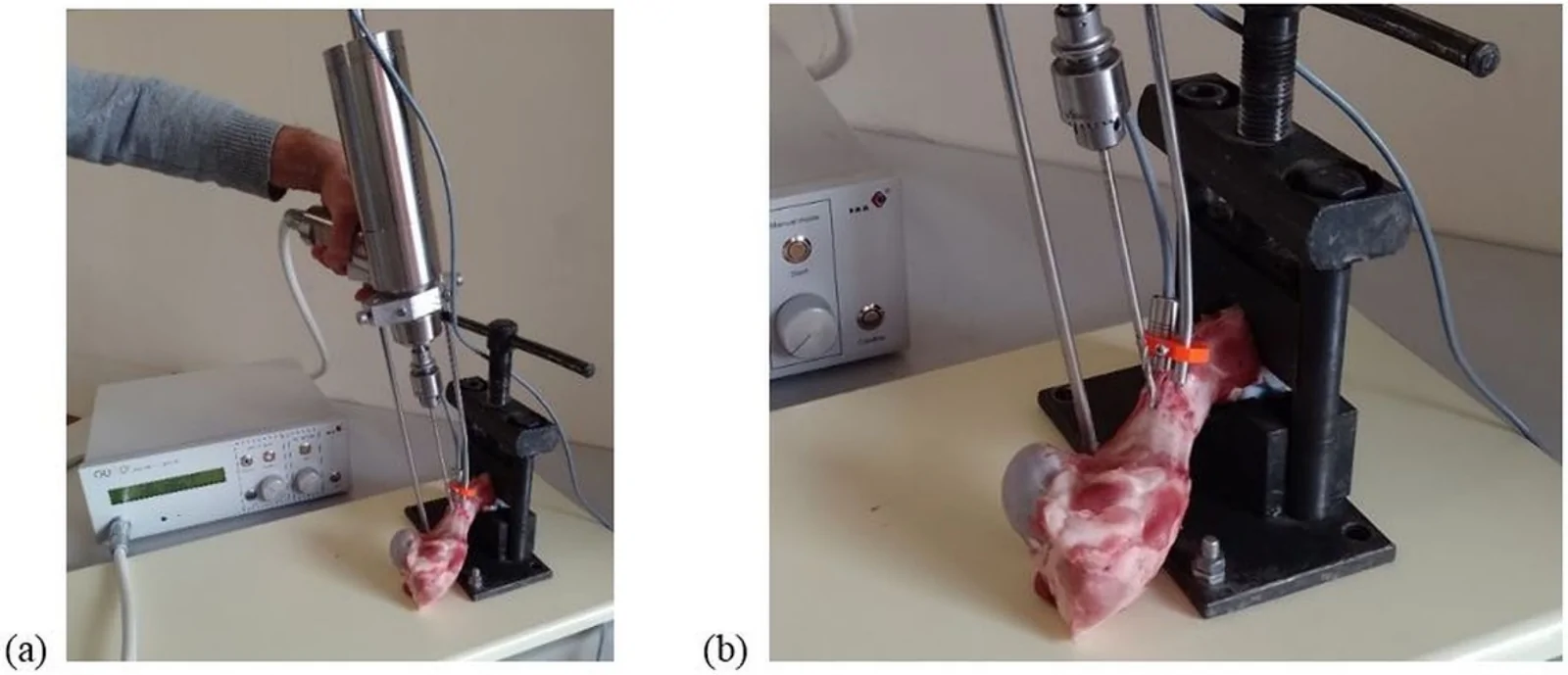
(a) - Experimental setup with the ODRO robot. (b) - Experimental setup. Temperature sensor head - close-up.

**Figure 3:**
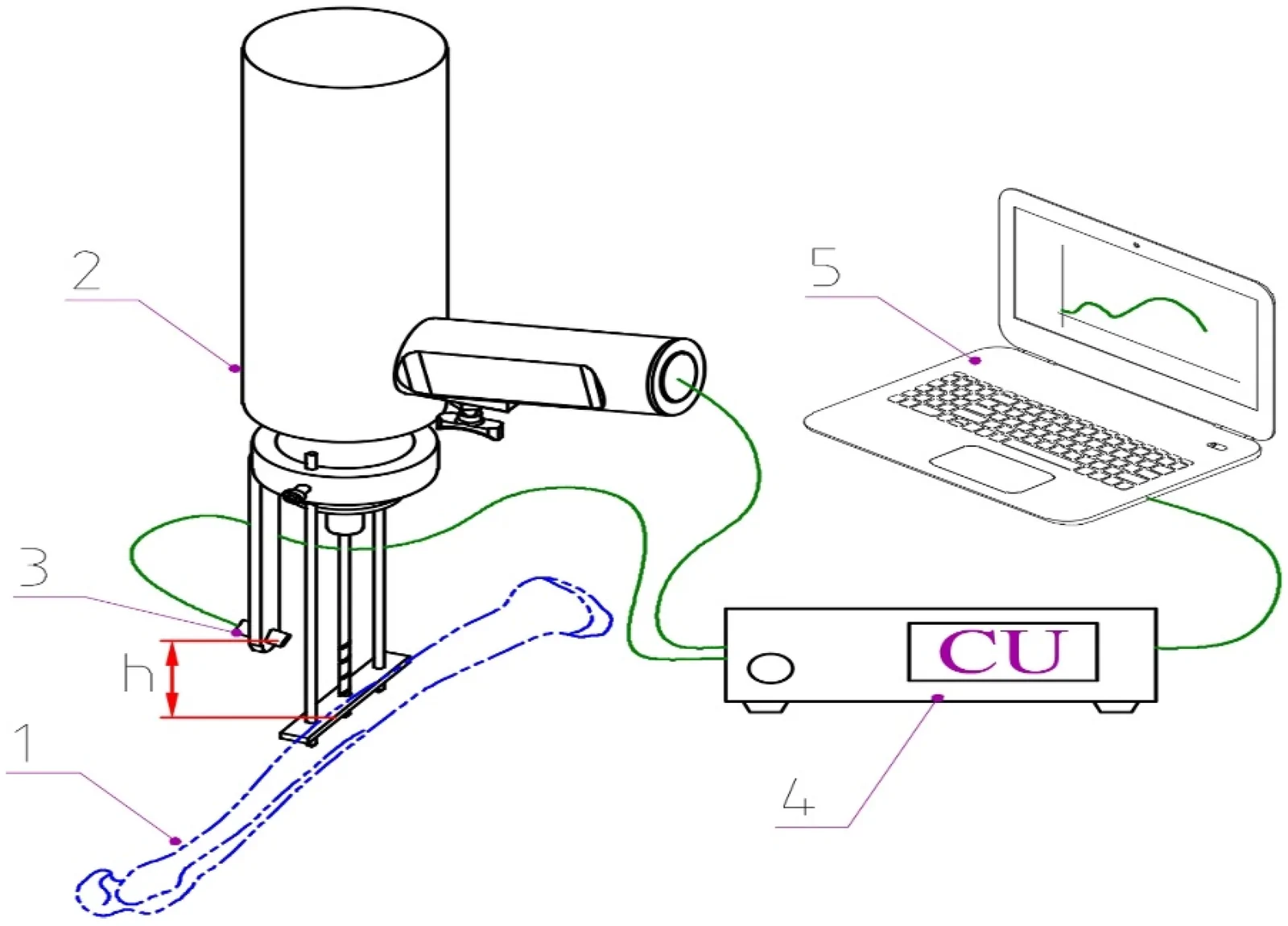
A schematic illustration of the experimental setup; 1. Bone specimen; 2. Drilling module; 3. Optical sensor head; 4. Control unit; 5. PC; h - Distance between the drill bit/bone contact point and the measurement point on the drill bit.

**Figure 4:**
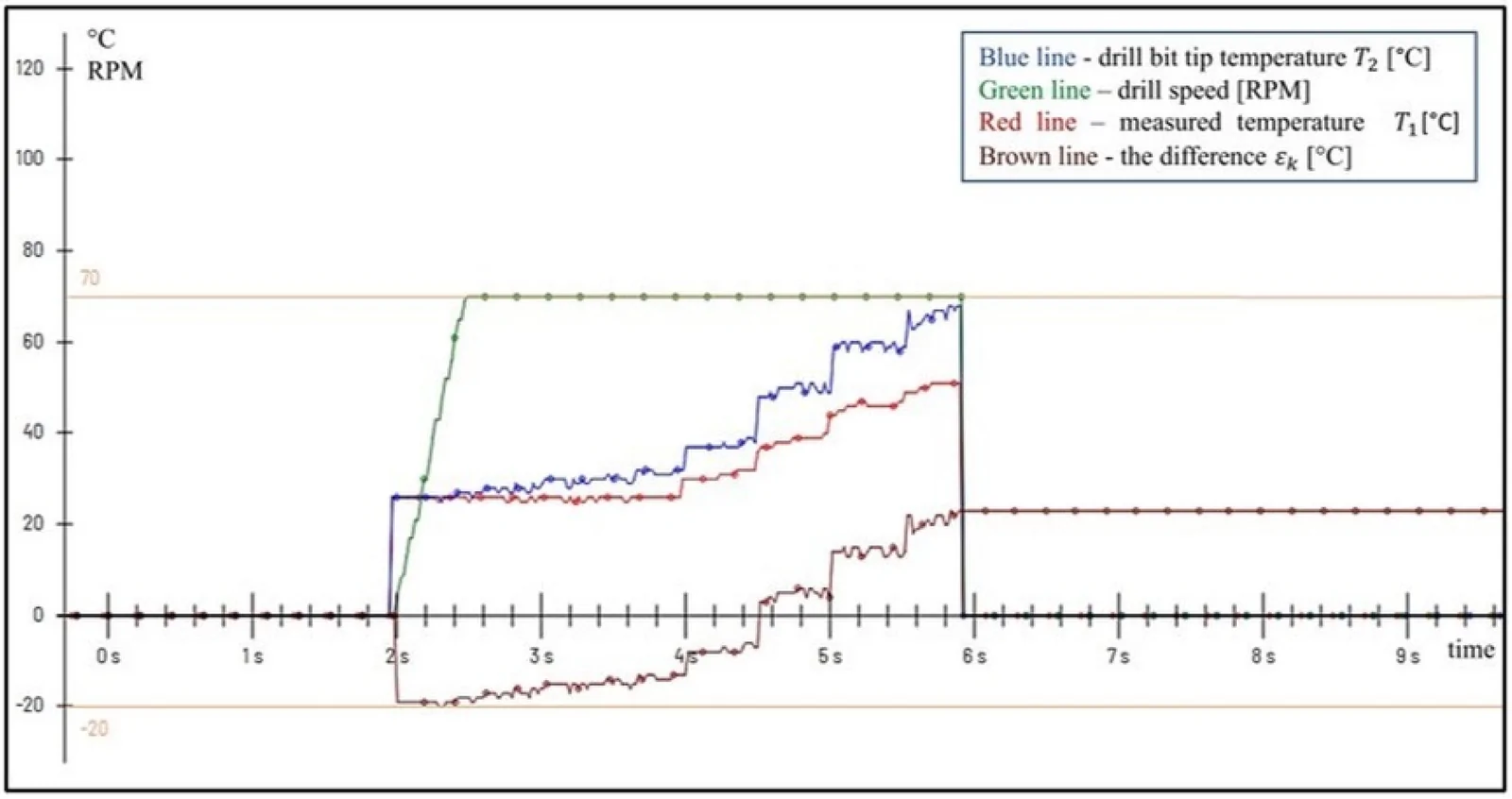
Unicortical drilling without drill speed control, NDB 4 mm and feed rate of 2mm/s.

**Figure 5:**
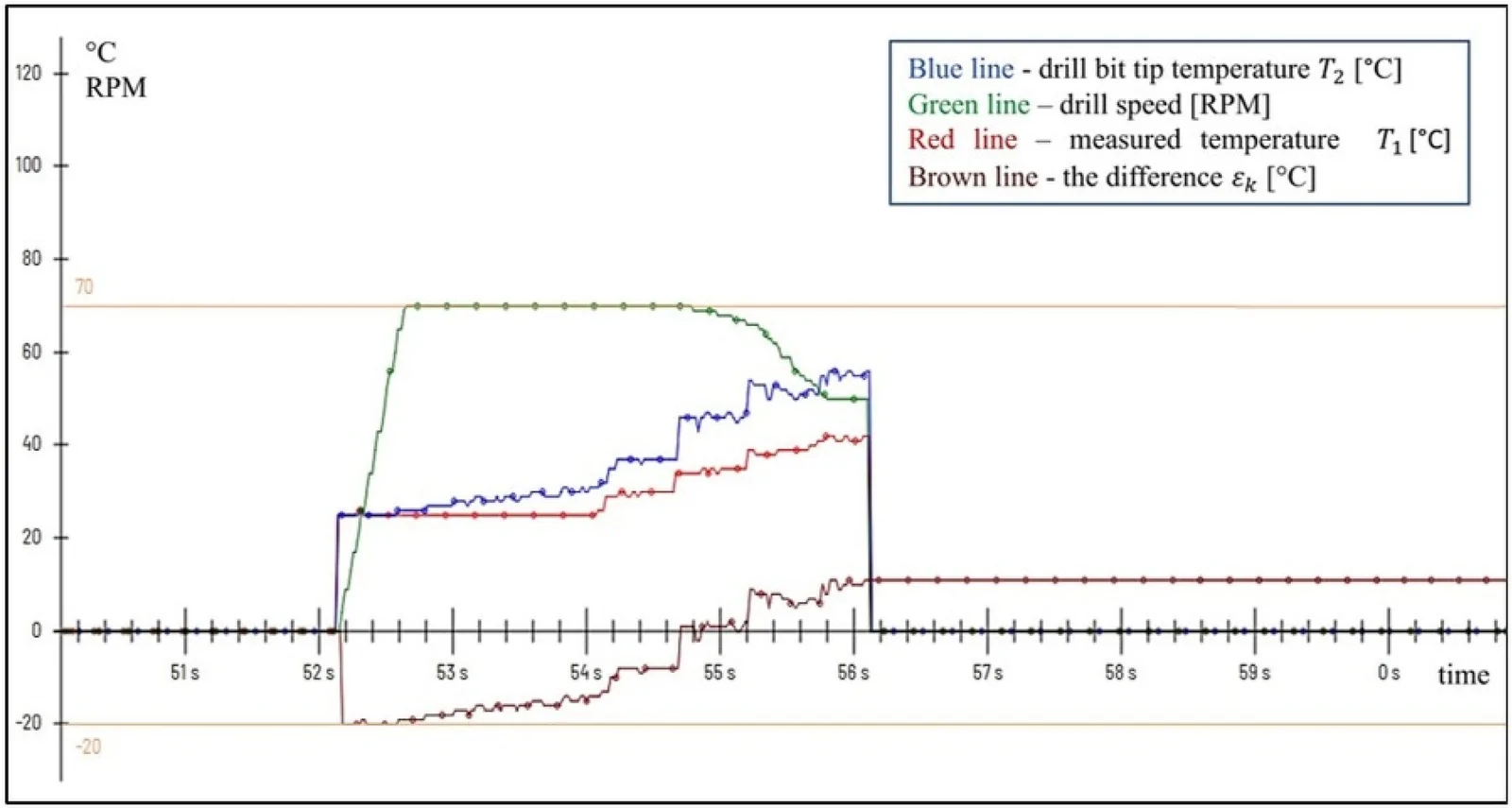
Unicortical drilling with drill speed control, NDB 4 mm and feed rate of 2mm/s.

**Figure 6:**
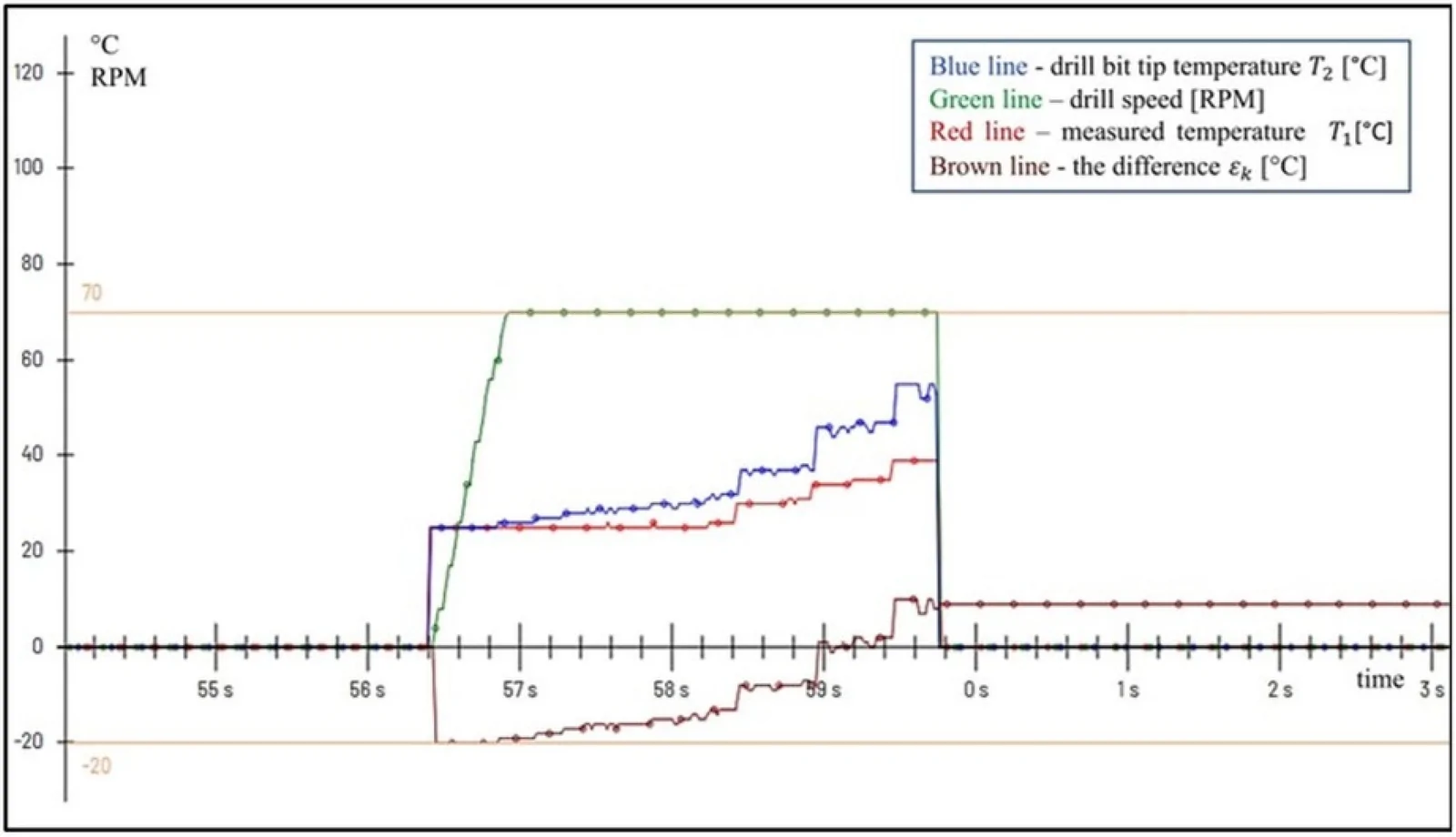
Unicortical drilling without drill speed control, NDB 3mm and feed rate of 2mm/s.

**Figure 7:**
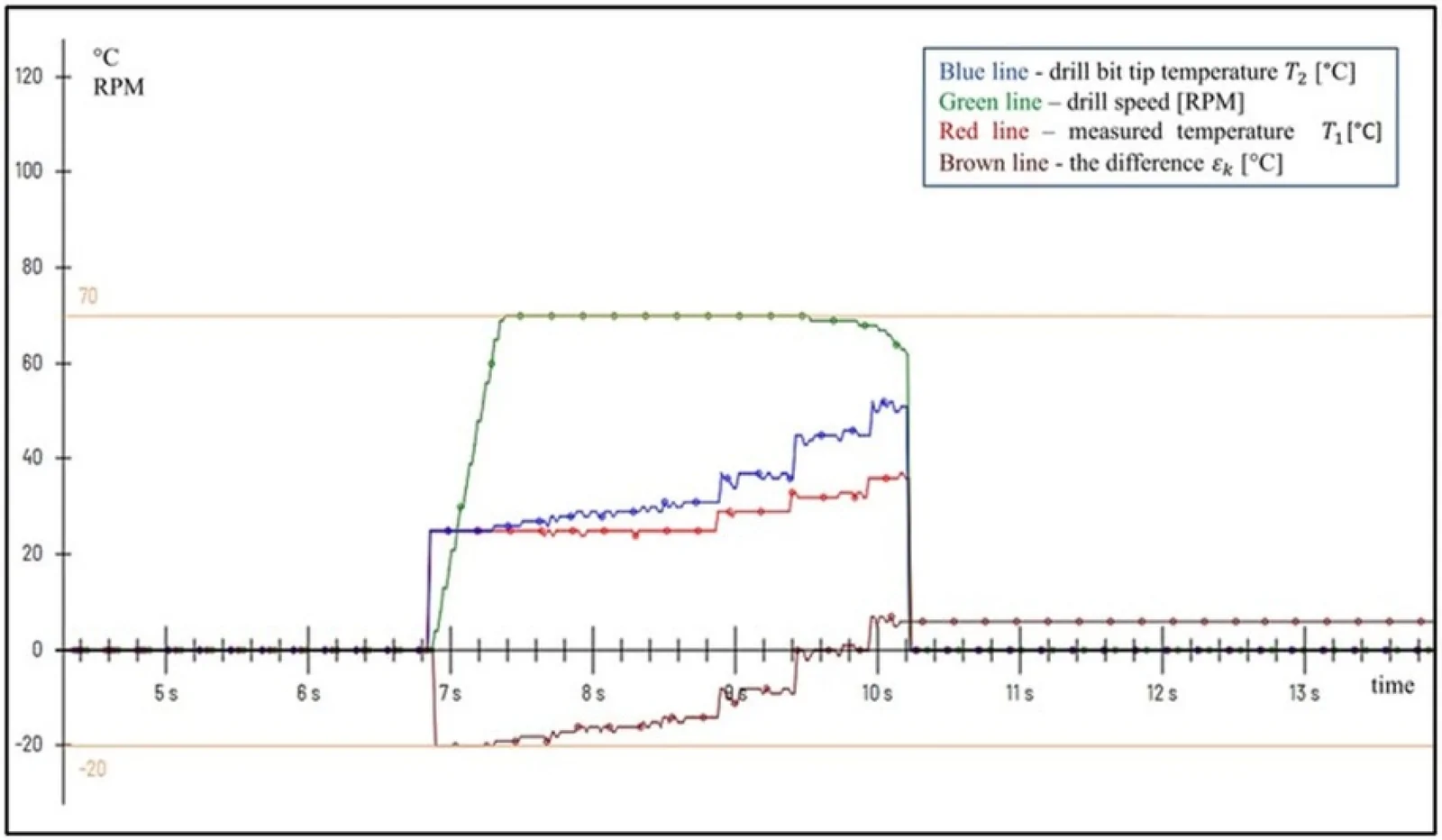
Unicortical drilling with drill speed control, NDB 3mm and feed rate of 2mm/s.

**Figure 8:**
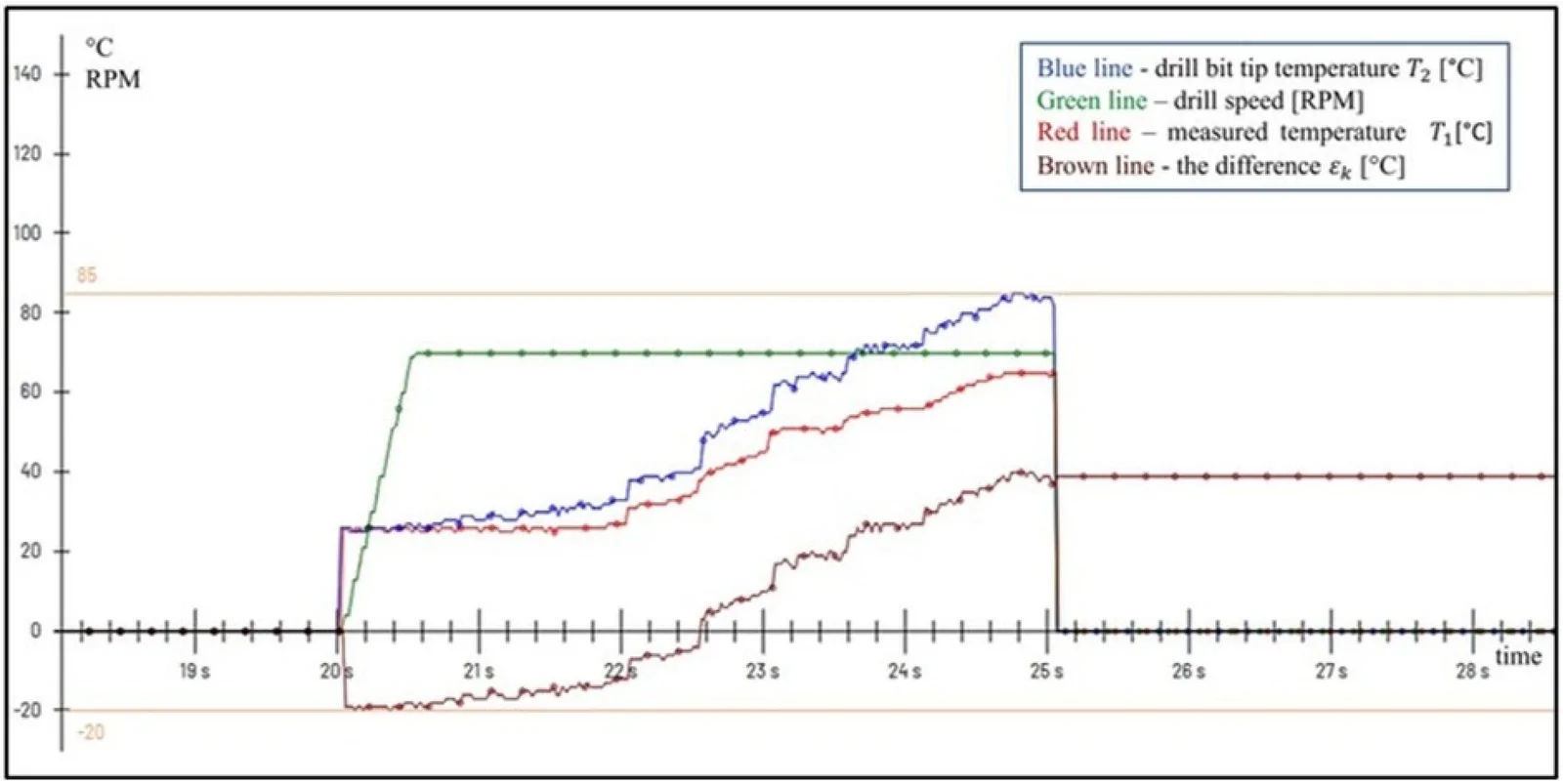
Unicortical drilling without drill speed control, UDB 3mm and feed rate of 2mm/s.

**Figure 9:**
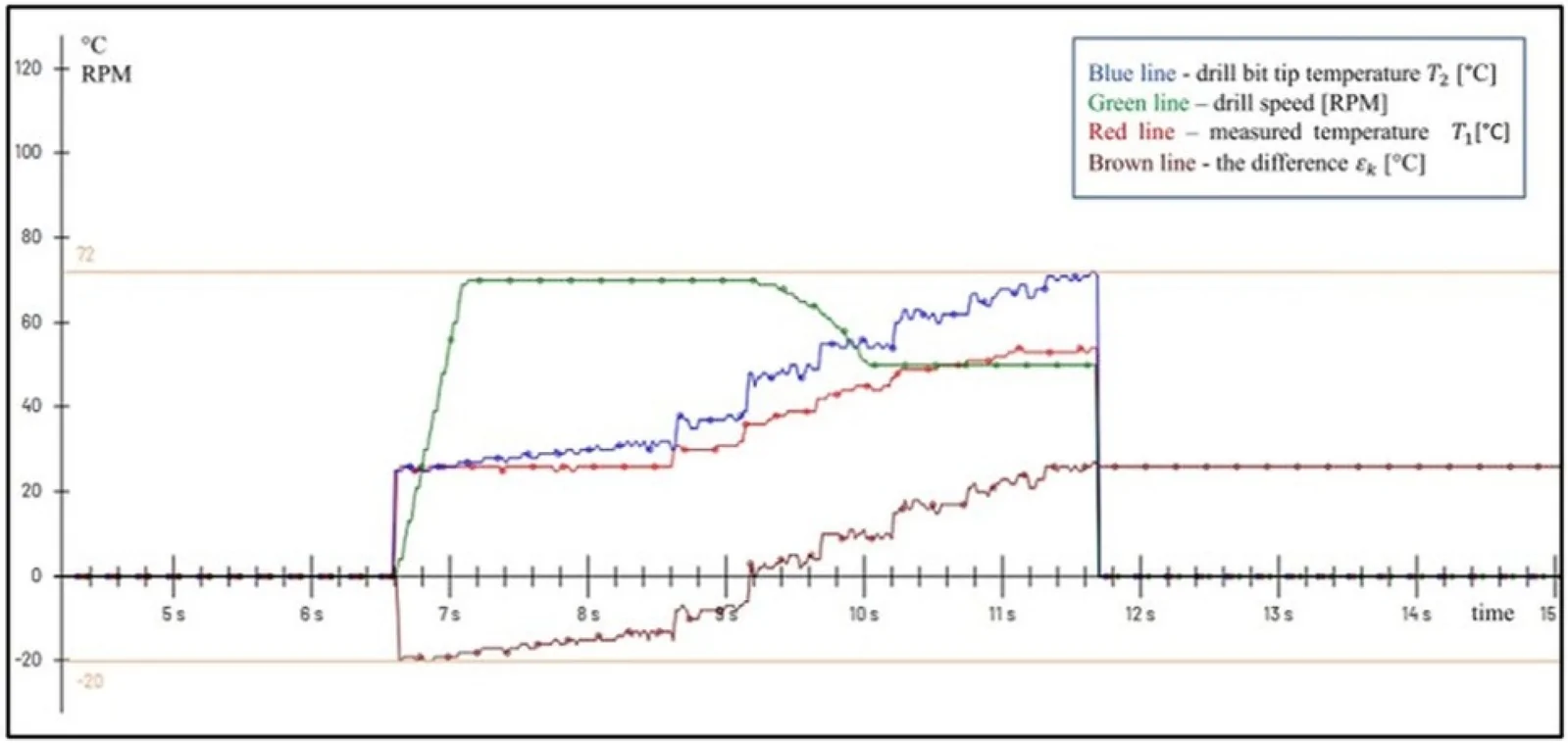
Unicortical drilling with drill speed control, UDB 3mm and feed rate of 2mm/s.

**Figure 10:**
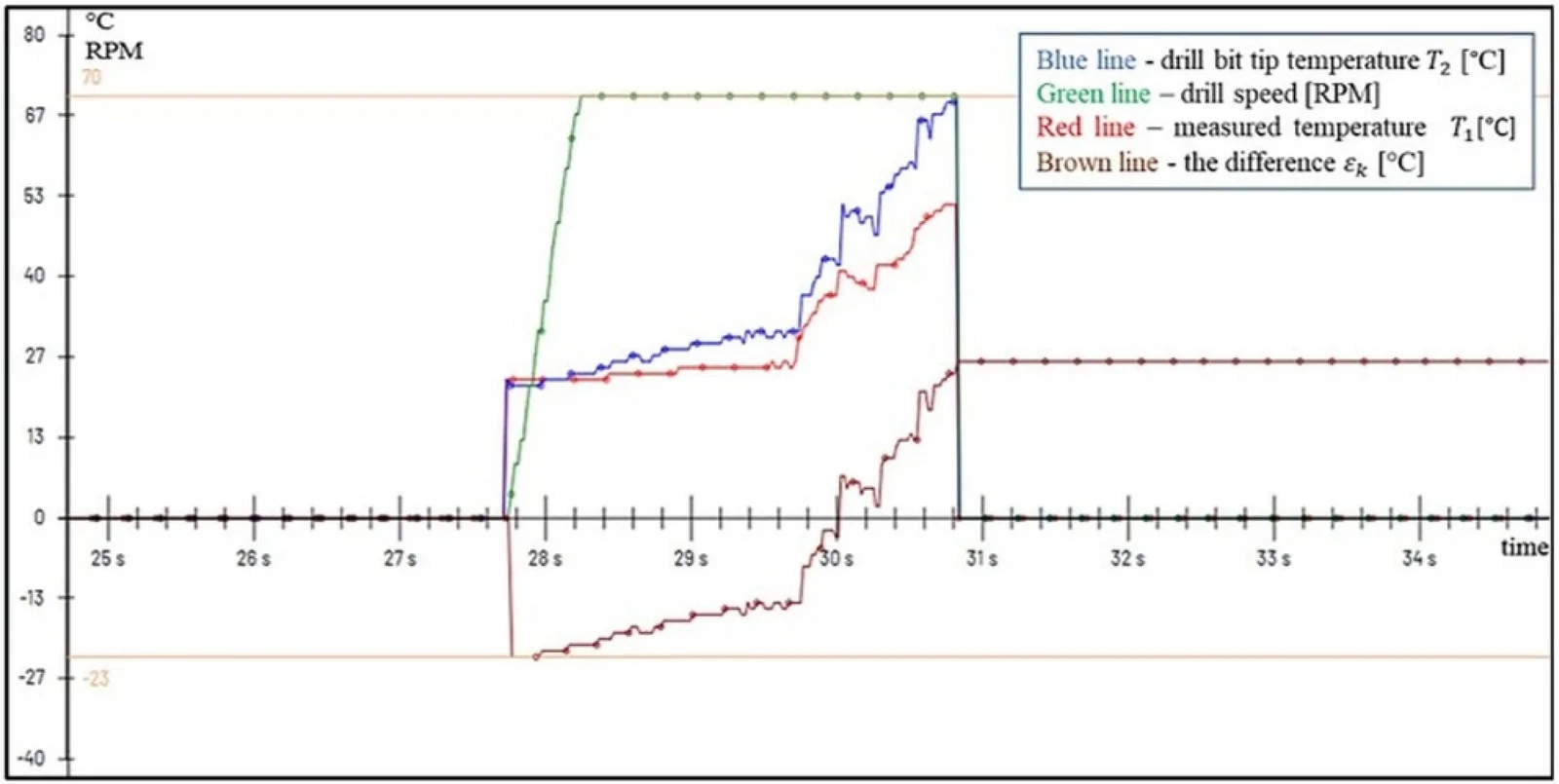
Unicortical drilling without drill speed control, VUDB 3 mm and feed rate 4mm/s.

**Figure 11:**
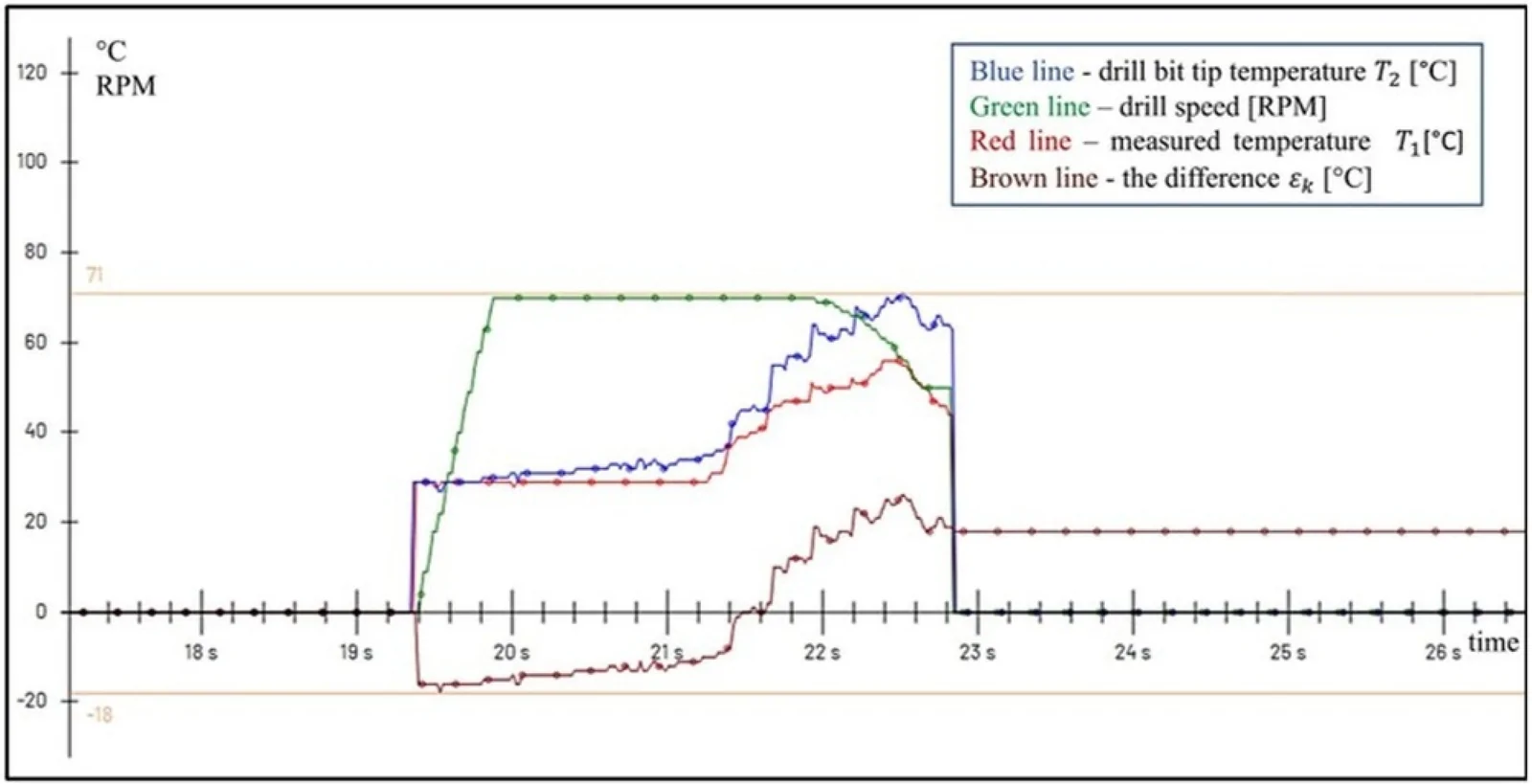
Unicortical drilling with drill speed control, VUDB 3 mm and feed rate 4mm/s.

**Figure 12:**
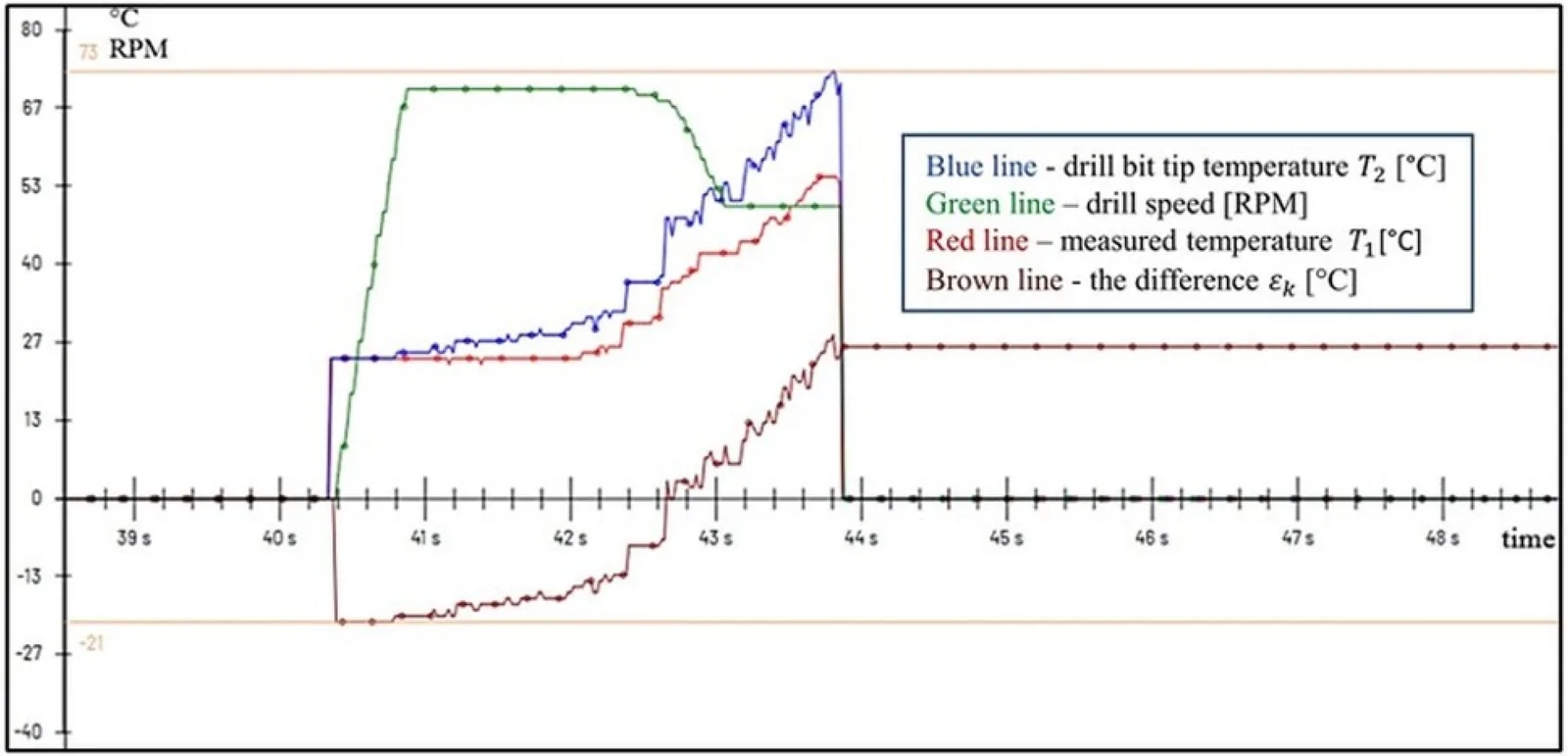
Unicortical drilling with drill speed control, WDB 3 mm, feed rate 4mm/s.

**Table 1:** Energy spaces and their basic variables, notations, descriptions, units of measurement, and expressions in basic units in the SI system

Energy Space	Notation		Description		Measure	Power	
Base Space	Across	Through	Terms	Unit	Dimension	P=XY	
Electric **H _EL_**	**U**	**I**	Voltage Current	Volt Ampere	L^2^MT^-3^ I^-1^ I	**UI**	L^2^M/T^3^
Magnetic **H _MG_**	**h**	**θ**	Magnetic field Intensity Induction flow velocity	Ampere/ meter Veber. meter/ second	L^-1^I L^3^MT^-3^ I^-1^	**h θ**	L^2^M/T^3^
Translation **H _TR_**	**v**	**F**	Linear velocity Force	Meter/ second Newton	LT^-1^ LMT^-2^	**v F**	L^2^M/T^3^
Rotation **H _R_**	**ω**	**T**	Angular velocity Torque	Rad/second Newton.meter	T^-1^ L^2^MT^-2^	**ωT**	L^2^M/T^3^
Hydraulic **H _HD_**	**P**	g*	Pressure Fluid flow	Newton/meter^2^ Meter^3^/second	L^-1^MT^-2^ L^3^T^-1^	P g*	L^2^M/T^3^
Thermal **H _T_**	***τ***	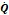	Temperature Thermal flow	Degree Joule/second	No Dimension L^2^MT^-3^	***τ*** 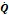	L^2^M/T^3^
Electrostatic **H _ES_**	e	q*	Electrostatic field Intensity Electric charge velocity	Volt/ meter Couomb.meter/second	LMT^-3^ I^-1^ TI.LT^-1^	e q^*^	L^2^M/T^3^

**Table 2:** Experimental results

№ of Fig.	Cortical thickness [mm]	Drill bit type [mm]	Feed rate [mm/s]	Drill speed control	Max T_2_ [ºC]	Temper. Limitation [ºC]	Total time [s]	Time over 60ºC [s]	Risk
4	4.8	NDB 4	2	no	68		4	1	no
5	4.8	NDB 4	2	yes	56	12	4	-	no
6	3.6	NDB 3	2	no	55		3.4	-	no
7	3.6	NDB 3	2	yes	51	4	3.4	-	no
8	5	UDB 3	2	no	85		5.1	2	yes
9	5.1	UDB 3	2	yes	72	13	5.1	1.4	no
10	3.8	VUDB 3	4	no	70		3.1	0.5	no/yes
11	4.4	VUDB 3	4	yes	71	7	3.4	0.9	no
12	3.5	WDB 3	4	yes	73		3.5	0.4	no/yes

## Data Availability

The datasets generated and/or analyzed during the current study are available from the corresponding author on reasonable request.
